# 气管镜治疗187例次因恶性肿瘤引起的阻塞性肺不张的临床分析

**DOI:** 10.3779/j.issn.1009-3419.2011.08.04

**Published:** 2011-08-20

**Authors:** 洪武 王, 冬妹 李, 楠 张, 珩 邹, 云芝 周, 晶 李, 素娟 梁

**Affiliations:** 100028 北京，煤炭总医院肿瘤微创治疗中心 Minimal Invasive Tumor Therapy Center, Meitan General Hospital, Beijing 100028, China

**Keywords:** 肿瘤, 气管镜, 介入治疗, Neoplasms, Bronchoscopy, Cryosurgery

## Abstract

**背景与目的:**

阻塞性肺不张往往伴有肺部感染和低氧血症等。尽快消除气道梗阻成为阻塞性肺不张的首要治疗目的。本研究旨在探讨气管镜治疗因恶性肿瘤引起的阻塞性肺不张的有效性和安全性。

**方法:**

回顾性分析2006年3月21日-2011年3月3日经病理证实的120例因恶性肿瘤引起的阻塞性肺不张病例，年龄5岁-90岁，均在气管镜下行氩等离子体凝固（argon plasma coagulation, APC）、冷冻等治疗。

**结果:**

120例患者合并肺不张187个，缘于原发肿瘤引起者98个，转移肿瘤引起者89个。原发气道肿瘤组和转移组肺不张最常见的部位分别为双上肺和单全肺。气管镜对两组气道内肿瘤的清除率相似，但原发肿瘤组肺复张率明显低于转移组。治疗后患者的体质评分（karnofsky physical score, KPS）均有明显升高，气促评分（shortbreath scale, SS）明显降低。术中约3/4出现低氧血症，3.4%发生术中大出血，其中1例死亡。大多数濒危的患者经气管镜治疗后生存期延长，中位生存时间为6个月，1年生存率为27.1%。

**结论:**

气管镜治疗能快速、有效清除气道内肿瘤，使阻塞的肺复张。

## Abstract (en)

**Background and objective:**

Obstructive atelectasis is frequently accompanied by pulmonary infection and hypoxia. The key to treating this condition is by directly reopening the obstructive airway. The aim of the present study is to explore the safety and efficacy of interventional bronchoscopy for the treatment of malignant obstructive atelectasis.

**Methods:**

A total of 120 cases with pathology-proved malignant obstructive atelectasis were retrospectively analyzed for the treatment of argon plasma coagulation and cryosurgery under bronchoscopy. Patients' age is between 5 and 90 years old.

**Results:**

A total of 120 cases had 187 atelectasis originating from 98 lesions with primary airway tumors and 89 with metastases. The most common location of atelectasis was in the upper lobe in the primary group and in the single lung in the metastasis group. Although there was no significant difference in tumor debulging between the two groups, the reopening rate of atelectasis was lower in the primary group than that in the metastasis group. The Karnofsky physical score significantly increased, and shortbreath scale decreased after interventional bronchoscopy. Among the patients, 3/4 had hypoxemia and 3.4% had severe bleeding, which caused the death of 1 patient during a procedure. The mean survival time was 6 months, and the survival rate of 1 year was 27.1%.

**Conclusions:**

Bronchoscopy can rapidly and effectively debulge the airway tumor and reopen the atelectasis.

恶性肿瘤是阻塞性肺不张最常见的病因^[1, 2]^，气管镜检查对明确阻塞的病因有重要价值，已成为肺不张必不可少的诊断工具。阻塞性肺不张往往伴有肺部感染和低氧血症等。因此，尽快消除气道梗阻成为阻塞性肺不张的首要目的。由于因恶性肿瘤引起的阻塞性肺不张大多处于肿瘤晚期，失去手术治疗时机，而放疗、化疗效果欠佳，所以气管镜介入治疗成为解除气道梗阻快速、有效的治疗手段^[3, 4]^。本文旨在回顾性分析作者近年来用气管镜治疗的120例因恶性肿瘤引起的阻塞性肺不张病例情况，以供临床参考。

## 1 资料与方法

### 1.1 临床资料

回顾性分析2006年3月21日-2011年3月3日间北京煤炭总医院收治的因恶性肿瘤引起的阻塞性肺不张共患者120例，年龄5岁-90岁，平均年龄（60.6±1.4）岁。其中男性96例，平均年龄（60.7±1.6）岁；女性24例，平均年龄（59.7±3.3）岁。肺部恶性肿瘤111例，包括鳞癌65例，腺癌17例，小细胞肺癌10例，黏液表皮样癌5例，腺鳞混合癌4例，腺样囊性癌、肌纤维母细胞瘤、乳头状癌、小细胞癌鳞癌混合癌各2例，基底细胞样癌和肉瘤各1例；其它器官转移而来的肿瘤9例，包括肾癌5例、大肠癌2例、肝癌和甲状腺癌各1例。根据TNM分期，Ⅰb期和Ⅱa期各2例，Ⅱb期6例，Ⅲa期12例，Ⅲb期36例，Ⅳ期62例。

根据来源不同，气道内肿瘤分为原发性和继发性。如果肿瘤原发于气道则为原发性气道肿瘤，本组98例肺不张是由原发气道内肿瘤引起；如果为非肺源性的肿瘤，并可追溯到异位原发灶则为远处转移，如果为肺源性也可根据病理或不同的病变部位来确定是否为转移瘤，本组89例肺不张由转移性肿瘤引起。

### 1.2 治疗方法

治疗方案经医院伦理委员会同意，并经患者本人和家属签署知情同意书。

#### 1.2.1 气管镜及配套设备

##### 1.2.1.1 电子支气管镜（简称软镜）

所用软镜为日本PENTAX-EPM3500。按电子支气管镜操作常规进行，术前给予无痛镇静及局部喷射麻醉^[5]^，术中持续静脉镇静麻醉。

##### 1.2.1.2 硬质镜

所用硬质镜为德国Karl Storz（Tutlingen）。操作在手术室进行。麻醉前面罩吸氧，预氧合5 min-10 min。术前10 min静脉滴注阿托品0.5 mg或东莨菪碱0.3 mg抑制气道内过多的分泌物。术中需监测血氧饱和度、心电图、血压及呼吸运动等。患者诱导前5 min应用咪哒唑仑2 mg静注，随后静注芬太尼（1-2）μg/kg，1%异丙酚（1-2）mg/kg。然后给予肌松剂阿曲库铵0.5 mg/kg，待肌颤消失、下颌肌肉松弛后即可经口插入硬质镜。维持药物浓度为1%异丙酚（1-2）mg/kg•h^-1^，瑞芬太尼（0.1-0.2）μg/kg•min^-1^。然后接麻醉呼吸机及高频喷射通气，通过硬质镜后端的操作孔进行各种操作^[6]^。

#### 1.2.2 气管镜介入治疗

氩等离子体凝固（argon plasma coagulation, APC）所用设备为德国产CESEL 3000型。将APC探针通过电子支气管镜活检孔伸出气管镜插入端（能见到探针标志为准），在距病灶0.5 cm以内时开始烧灼。APC输出功率为30 W-50 W，氩气流量为（0.8-1.6）L/min。烧灼过程中勿需停止吸氧，但以间断烧灼为宜，每次5 s-10 s左右，时间不能太长，并不断用活检钳取出碳化凝固的组织（碳化的组织易燃着火）。

冷冻机采用北京库兰医疗设备有限公司生产的冷冻治疗仪K300型和德国ERBE。电圈套器型号为南京微创公司生产。被膜金属支架为江苏西格玛公司生产。软式可弯曲冷冻探头直径1.9 mm-2.3 mm，探针末端长度5 mm。冷源为液态二氧化碳。将冰冻探头的金属头部放在肿瘤表面或推进到肿瘤内，冷冻5 s-10 s，使其周围产生最大体积的冰球，在冷冻状态下将探头及其粘附的肿瘤组织取出，必要时再插入探头，直至将腔内的肿瘤全部取出。冻取后如有出血，则结合APC止血。若将冰冻探头的金属头部放在病灶表面持续冷冻1 min-3 min，称为冻融。

电圈套器型号为南京微创公司生产。将电圈套器连接在高频电刀上。通过电子支气管镜的活检通道将电圈套器套扎在肿瘤上，然后启动高频电凝将肿瘤切下，再用光学活检钳或冷冻将切下的肿瘤取出。

#### 1.2.3 疗效判断方法

##### 1.2.3.1 气道狭窄程度

$
气道狭窄(\% ) = \frac{{正常气道宽度-最狭窄处的气道病变宽度}}{正常气道宽度} \times 100\% 
$

##### 1.2.3.2 气道内肿瘤的疗效判断

完全缓解（complete response, CR）：气道内肿瘤完全消除，气道无狭窄；部分缓解（partial response, PR）：气道内肿瘤部分消除，气道狭窄≤50%；无效（no response, NR）：气道内肿瘤大部分未消除，气道狭窄 > 50%。

##### 1.2.3.3 肺复张程度的疗效判断

肺完全复张（complete reopening, CR）：—肺完全膨胀，体积恢复正常，无任何肺不张残留；肺部分复张（partial reopening, PR）：—肺部分膨胀，体积较前增大，膨胀的部分肺纹理可见，未复张的部分仍为体积缩小的致密影；肺复张无效（no reopening, NR）：—肺体积未变，仍为肺不张的致密影。

##### 1.2.3.4 体质评分（karnofsky physical score, KPS）和气促评分（shortbreath scale, SS）

根据文献^[7]^在气管镜介入治疗前和治疗后第2天进行评价、记录。

### 1.3 统计学处理

采用SPSS 11.0统计软件包进行分析，分类资料采用χ^2^检验，计量资料采用*t*检验或非参数检验，以*P* < 0.05为有统计学差异。

## 2 结果

### 2.1 气管镜介入治疗对气道内肿瘤的消除及肺不张的改善情况

120例患者合并阻塞性肺不张187个（来自原发肿瘤引起者98个，转移性肿瘤引起者89个），共进行气管镜操作174例次，其中气管插管6例次，喉罩2例次，软镜71例次，硬镜95例次。

由表 1可见，在原发气道肿瘤引起的肺不张患者中两肺发生肺不张的次数相似，但双上肺最为常见（占44.9%，χ^2^=27.918，*P* < 0.001，右上肺最多，其次为左上肺），其次为全肺不张（16.3%），左全肺不张明显多于右全肺不张（15.3% *vs* 1%, χ^2^=1.338, *P* < 0.001）。41.8%（41/98）的气道内肿瘤可经气管镜治疗完全消除（图 1），有效率（CR + P R）达9 8 %。以右中叶支气管的疗效最佳。经气管镜治疗后，12.2%（12/98）可完全复张，部分复张率达17.3%（17/98），有效率达29.6%（29/98）。左肺复张的有效率高于右肺（43.7% *vs* 16%, χ^2^=9.051, *P* < 0.05）。

**1:**
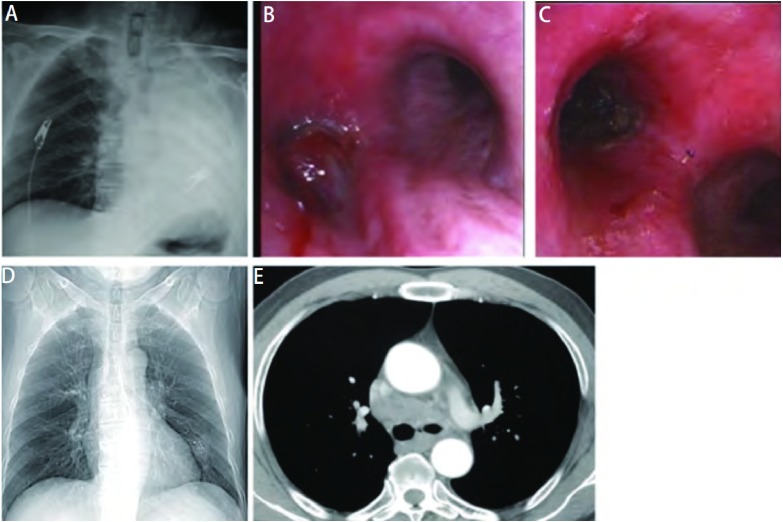
原发左主支气管肿瘤引起的左全肺不张气管镜治疗效果（男，57岁）。A：胸片示左全肺不张，气管左偏；B：气管镜示左主支气管开口被肿瘤完全阻塞；C：经气管镜治疗后左主支气管开口通畅，肿瘤被基本清除，术后病理为鳞癌；D：胸片示左肺已完全复张，双侧支气管通畅，左下肺有一肿块，内有高密度影（已植入放疗粒子）；E：胸部CT示左肺已完全复张，双侧支气管通畅，纵隔淋巴结肿大。 The bronchoscopic effect of whole left etelectasis in patient with primary left bronchus carcinoma (Male, 57 years old). A: Chest film: Whole left etelectasis with trachea moving to left lung; B: Brconchoscopy: The orifice of left main bronchus was completely obstructed by a tumor; C: The squamous carcinoma in left main bronchus was completely removed by bronchoscopy, and the orifice of left main bronchus was successfully reopened; D: Chest film: The whole left etelectasis disappeared after interventional bronchoscopy. The radioactive seeds were discovered in left lower lung mass; E: Computer tomography: The whole left etelectasis disappeared, and bilateral bronchus was normal, but mediastinal lymphonode enlargement was discovered.

**1:** 气管镜治疗对98例原发气道肿瘤引起的肺不张的疗效 The effect of interventional bronchoscopy for the treatment of atelectasis in 98 patients with primary airway tumors

Obstructive location	*n*	Clearance of airway tumor				Reopening of atelectasis		
		CR^a^	PR^b^	NR^c^		CR^d^	PR^e^	NR^f^
Right bronchus								
Right main bronchus	1	0	1	0		0	1	0
Right upper bronchus	25	14	11	0		1	2	22
Right Inermedius bronchus	11	3	7	1		0	2	9
Right Middle bronchus	8	6	2	0		0	0	8
Right lower bronchus	5	0	5	0		0	2	3
Left bronchus								
Left main bronchus	15	6	8	1		4	2	9
Left upper bronchus	19	6	13	0		4	5	10
Left lower bronchus	14	6	8	0		3	3	8
Total	98	41	55	2		12	17	69
CR^a^: complete reopense; PR^b^: partial reopense; NR^c^: no reopense; CR^d^: complete reopening; PR^e^: partial reopening; NR^f^: no reopening.								

由表 2可见在转移性气道肿瘤引起的肺不张患者中两肺发生肺不张的次数相似，但以两全肺不张最为常见（占49.4%，左肺多于右肺），明显高于原发肿瘤引起的全肺不张（χ^2^=23.767, *P* < 0.01）。其次为双上肺不张占25.8%，但低于原发肿瘤引起的44.9%的双上肺不张（*t*=1.617, *P* > 0.05）。46.1%（41/89）的气道内肿瘤可经气管镜治疗完全消除（图 2），有效率（CR+PR）达97.7%，但各段支气管内肿瘤的消除率相似。经气管镜治疗后CT复查发现肺完全复张率达12.3%（11/89），部分复张率达43.8%（39/89），有效率达56.2%（50/89），明显高于原发肿瘤引起的肺不张的疗效（χ^2^ = 1 3. 5 4 1, *P* < 0.01）。双肺复张的有效率相似。

**2:**
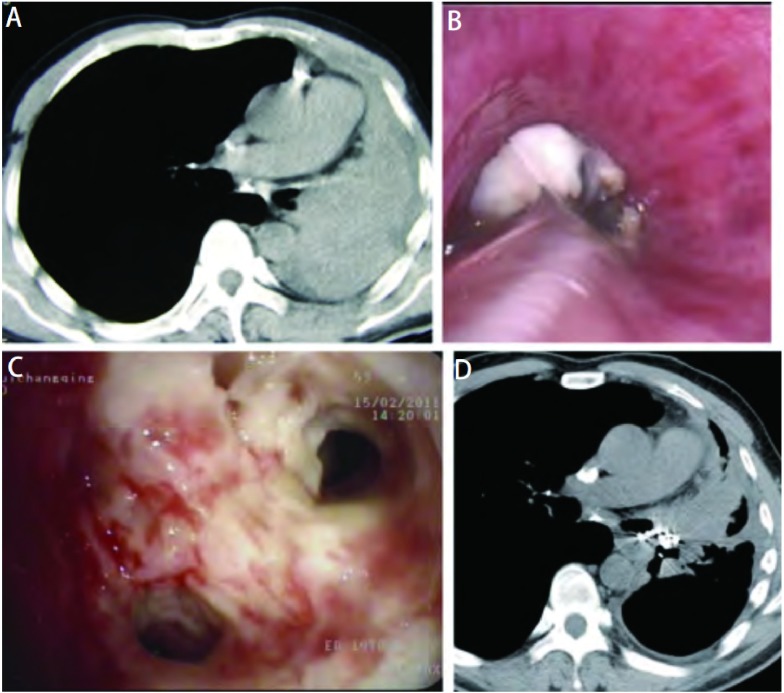
左上肺基底细胞样癌引起的左全肺不张（男，52岁）。A：胸部CT示左全肺不张，左主支气管内可见占位性病变；B：气管镜示左主支气管中段被表面有坏死物的肿瘤完全阻塞；C：经气管镜治疗后左主支气管内肿瘤清除，左下叶基底段开口可见，肿瘤位于左上叶开口，已将左上叶完全阻塞，术后病理检查为基底细胞样癌；D：胸部CT示左下肺已完全复张，左上肺部分复张，左上肺内可见高密度影（植入的放射性粒子）。 Whole left lung atelectasis caused by basolid cellular carcinoma in left main bronchus (Male, 52 years old). A: Chest CT: Whole left lung atelectasis caused by occupation in left main bronchus; B: Bronchoscopy: The middle segment of left main bronchus was completely obstructed by a tumor with necrosis; C: Bronchoscopy: The tumor was completely removed after bronchoscopic treatement, but the left upper segment is still obstructed from which is the tumor originated. The left lower segments were reopened after interventional treatment. The basolid cellular carcinoma was pathologically proved by tumor biopsy; D: Chest CT: Left lower lung was reopened completely and left upper lung was partially reopened. Radioactive seeds were implanted in left upper lung.

**2:** 气管镜介入治疗对89例转移性气道内肿瘤的消除及肺不张的改善情况 The effect of interventional bronchoscopy for the treatment of atelectasis in 87 patients with metastatic airway tumors

Obstructive location	*n*	Clearance of airway tumor				Reopening of atelectasis		
		CR^a^	PR^b^	NR^c^		CR^d^	P^e^	NR^f^
Right bronchus								
Right main bronchus	17	12	5	0		3	7	7
Right upper bronchus	12	4	8	0		3	1	8
Right inermedius bronchus	9	4	5	0		1	6	2
Right middle bronchus	1	1	0	0		0	1	0
Right lower bronchus	5	1	4	0		0	2	3
Left bronchus								
Left main bronchus	27	12	15	0		4	16	7
Left upper bronchus	11	5	4	2		0	1	10
Left lower bronchus	7	2	5	0		0	5	2
Total	89	41	46	2		11	39	39
CR^a^: complete reopense; PR^b^: partial reopense; NR^c^: no reopense; CR^d^: complete reopening; PR^e^: partial reopening; NR^f^: no reopening.								

### 2.2 气管镜介入治疗对肺不张患者临床的改善情况

由表 3可见，气管镜介入治疗前以右全肺不张（右主支气管阻塞）患者KPS最低，SS最高，而右中叶不张KPS最高，SS最低；治疗后肺不张患者KPS和SS除右中叶不张改善不明显外，其它各组均有明显改善，以全肺不张组改善最为明显。

**3:** 气管镜介入治疗对肺不张患者KPS及气促评分的影响 Changes of KPS and shortbreath scale for interventional treatment of bronchoscopy in patients with atelectasis

Obstructive location	*n*	Karnofsky physical score			Shortbreath scale	
		Before	After		Before	After
Right bronchus						
Right main bronchus	18	47.2±3.8	71.1±4.1^*^		3.6±0.2	1.9±0.2^*^
Right upper bronchus	35	54.4 ±3.5	75.1±2.3^*^		2.8±0.1	1.6±0.1^*^
Right inermedius bronchus	20	8.1±3.8	73.7±3.3^*^		2.7±0.3	1.4±0.2^*^
Right middle bronchus	9	71.2±8.5	85.0±4.6		2.5±0.4	1.9±0.1
Right lower bronchus	10	48.0±6.8	73.0±6.3^*^		2.9±0.3	1.6±0.2^*^
Left bronchus						
Left main bronchus	42	51.8±2.7	70.9±2.2^*^		3.2±0.1	1.9±0.2^*^
Left upper bronchus	24	56.7±3.8	74.2±3.2^*^		2.7±0.2	1.7±0.2^*^
Left lower bronchus	21	66.5±3.3	83.1±3.2^*^		2.7±0.2	1.4±0.2^*^
^*^Comparison between before and after treatment, *P* < 0.01.						

### 2.3 并发症

174次操作中发生术中大出血（> 100 mL）6例次（占3.4%），其中1例术中死亡（死亡率0.6%）。大出血时切勿退出气管镜，应持续负压吸引，局部注射止血药（巴曲酶、肾上腺素），必要时同时静注止血药。应变换体位，卧向患侧，以免血液灌注健侧肺。在用APC止血的过程中74.5%的患者会发生低氧血症，同时出现窦心动过速，经停止APC操作及吸氧后大多很快恢复正常。无1例发生着火或管壁穿孔等并发症。术后并发症主要是坏死物阻塞。经APC处理后，可产生坏死物，附着在创面上，如不及时清理，可能引起气道狭窄或梗阻，一般术后需给予雾化吸入，3天-7天需再次复查气管镜，以清除坏死物质。肺复张后，肺内可排出大量痰液，术后需应用化痰药，协助将痰液排出。

### 2.4 随访情况

术后电话或门诊随访3个月-48个月。失访12例，随访率为90%。中位生存时间为6个月，1年存活率为27.1%。

## 3 讨论

肺不张是呼吸系统常见病，其主要的病理形态学改变为一侧、一叶或一段肺组织局部无气体，肺组织萎陷。因恶性肿瘤引起的阻塞致肺不张可分为一侧全肺不张、肺叶不张或肺段不张。阻塞性肺不张大多病变在大气道内，如主气管、主支气管、右中间段支气管和段支气管，与肺部病变不等同，可由气道原发性和转移性恶性肿瘤引起。

本组资料120例患者中111例为肺源性肿瘤（包括原发和转移），9例为远位转移而来。一个患者可以有多个病灶，引起多个部位的肺不张。120例患者合并肺不张187个，由原发肿瘤引起的肺不张为98个，转移瘤引起者为89个。原发气道肿瘤和转移性气道肿瘤引起肺不张的部位明显不同，前者以双上肺肺不张最为常见(占44.9%)，其次为全肺不张（16.3%），左全肺不张明显多于右全肺不张。在转移性气道肿瘤引起的肺不张患者中以全肺不张最为常见（49.4%），明显高于原发肿瘤引起的全肺不张（*P* < 0.01），其次为双上肺不张（25.8%）。

气管镜检查不但可明确肿瘤阻塞的部位，又可直接治疗。近年来随着气管镜介入治疗技术的发展，镜下消瘤治疗成为开通气道阻塞最有效、快速的手段。现代电视硬质镜的价值在于可作为介入通道允许软性支气管镜及其他器械进入气道内，大大拓宽了其应用范围，既可用于大气道病变的治疗，也可用于段支气管病变的治疗。通常是以硬质镜作为通道并保障通气，如果肿瘤位于主气管内，用各种硬质器械均可操作；如果肿瘤位于支气管内，最好结合电子支气管镜进行各种操作。

大气道恶性肿瘤引起的肺不张，肯定有大气道梗阻，气管镜介入治疗应作为首选。虽然不能延长生存期（与病情分期有关），但能明显改善生存质量，这也是晚期患者治疗的主要目的，让其在有限的生存时间内改善生活质量是最重要的。传统认为气道内肿瘤临床放化疗是首选，其实不然，如果有过硬的气管镜介入治疗技术，镜下治疗是最有效、最快速的治疗方法，应列为首选，然后再去做放疗、化疗。本组患者大多数已经过放疗、化疗，或病情较重而不能行放疗和化疗。

结果显示，无论是原发气道内肿瘤或是转移性气道内肿瘤，两组肿瘤的完全清除率、有效率和两组肺完全复张率均相似。但肺部分复张率和有效率前组明显低于后组。前组左肺复张的有效率高于右肺，而后组双肺复张的有效率相似。气道内肿瘤的清除率与肺不张不完全吻合，大气道内肿瘤基本可全部清除，但若有亚段支气管堵塞，肺仍难以复张。而对亚段肺不张则不必苛求再通，否则会损伤大血管引起出血。另外，亚段肺不张对肺功能的影响也不大，也没必要一定再通，后期可结合放疗、化疗等将残余肿瘤清除，阻塞的肺不张还可再通。

本文22例患者TNM分期为早中期病例，其中Ib期2例（1例为80岁鳞癌，1例为小细胞肺癌），2例均经气管镜将管腔内肿瘤全部清除，肺完全复张，后期结合放疗、化疗，避免了手术切除。IIa期2例均为黏液表皮样癌（低度恶性肿瘤）、IIb期6例（黏液表皮样癌3例，腺癌、鳞癌和肌纤维母细胞瘤各1例），8例患者经气管镜治疗后管腔内肿瘤亦全部清除，肺全部复张，6例低度恶性肿瘤术后未再进行放、化疗，均未复发。2例腺癌、鳞癌则术后结合化疗，随访1年未再复发。Ⅲa期共12例，其中4例发生全肺不张患者拒绝全肺切除，1例为小细胞肺癌，其余也因年龄较大（2例 > 80岁）等原因拒绝手术。但患者经气管镜姑息治疗后，肺不张1例达CR，8例PR，肺复张率达75%，3例NR（1例为全肺不张的患者，1例为右中下叶不张，1例左下叶背段不张）。因此，对早中期肺癌患者气管镜介入治疗同样可取得非常好的疗效，有些患者可避免手术。

从气管镜介入治疗的临床改善程度来看，治疗前以右全肺不张（右主支气管阻塞）患者KPS最低，SS最高，而右中叶不张KPS最高，SS最低；治疗后肺不张患者KPS和SS除右中叶不张改善不明显外，其它各组均有明显改善，以全肺不张组改善最为明显。实际上，经支气管镜介入治疗无严格的指征。在我院进行气管镜检查的同时即进行介入治疗，对镜下所见肿瘤行及时清除，没必要再等病理诊断。当然，气管镜治疗后还可根据病理进行手术、放疗、化疗等。大多数患者气管镜治疗后梗阻解除，则不愿再做手术治疗，或已经行放疗、化疗，患者体质较弱，不能再耐受手术。支气管镜介入治疗可反复进行，以保障气道通畅。

麻醉对保证手术顺利进行至关重要。作者在早期阶段主要采取局部麻醉、电子支气管检查的方法。由于肺不张需多次治疗，很多患者难以接受反复操作。监视下麻醉又称神经镇静安痛术，具有时间短暂、患者无痛苦记忆、恢复迅速等优点，近年来作者实施的90%以上的电子支气管介入治疗均采用此方法。对病情较重、不能配合的患者则在全麻下进行硬质镜检查。本组120例患者共进行气管镜操作174例次，其中71例次在监视下麻醉单纯用电子支气管镜，103例次则在全麻下应用硬质镜95例次、气管插管6例次、喉罩2例次。

许多阻塞性肺不张的患者难以平卧，在全麻下插入硬质镜，既可保证患者的通气，又可从容地进行各种操作。本文气管内采用的治疗方法有钳取法4例次、冻取法120例次、电圈套器10例次、APC 160例次、局部注药20例次、内支架5例。具体采用哪种方法最合适需考虑内镜技术的熟练程度、已有的设备条件等。对有蒂或瘤体较长的肿瘤适合用电圈套器或光学活检钳将肿瘤直接切除；对瘤体表面较脆、易出血的肿瘤则适宜先用APC封闭血管，再结合冷冻将肿瘤冻取（本组大部分采用此方法）；对瘤体较弥漫、不易出血的肿瘤，亦可直接用冻取的方法，必要时结合APC。作者在早期阶段主要采用APC及冷冻的方法，但由于术中出血较多（出血发生率占3.4%），风险较大，所以近年来作者不断改进治疗技术，采用电圈套法及先用APC后用冻取等方法使出血几率已大大减少，现在发生大出血的情况已较为少见。有些肿瘤血供丰富，如转移性肿瘤、腺样囊性癌等，术中需特别谨慎。本组只有1例术中因大出血死亡。

治疗过程中另一常见的并发症是低氧血症，APC治疗过程中几乎74.5%的患者会发生，因此，需间断应用APC，必要时使用麻醉机手动呼吸，使血氧饱和度维持在90%以上。低氧血症一般是短暂的、可逆的。只要不持续烧灼，一般不会发生严重的低氧血症。但若发生大出血，或瘤块脱落阻塞气道时，也可引起低氧血症，此时需及时将血块或肿块取出，低氧血症会很快纠正。

由于积极治疗大多数濒危的患者生存质量提高，KPS和SS均有明显改善, 生存期延长，中位生存时间为6个月，1年存活率为27.1%。但由于本组病例复杂，多种因素会影响患者预后，如患者的临床分期、病变部位及气管镜介入治疗前后所接受的治疗等，有待进一步进行分析。

总之，只要术中做好充分准备，备齐各种治疗设备，麻醉师密切配合，大部分气道内肿瘤均可在气管镜下完全清除，大部分肺不张也可复张，并且操作快速、有效、安全。
